# Parental Vaccine Hesitancy in Quebec (Canada)

**DOI:** 10.1371/currents.outbreaks.9e239605f4d320c6ad27ce2aea5aaad2

**Published:** 2016-03-07

**Authors:** Eve Dubé, Dominique Gagnon, Zhou Zhou, Genevieve Deceuninck

**Affiliations:** Institut National de Santé Publique du Québec, Québec, Canada; Centre de Recherche du CHU de Québec, Université Laval, Québec, Canada; Institut National de Santé Publique du Québec, Québec, Canada; Centre de Recherche du CHU de Québec, Université Laval, Québec, Canada; Centre de Recherche du CHU de Québec, Université Laval, Québec, Canada

## Abstract

Introduction: "Vaccine hesitancy" is a concept frequently used in the discourse around vaccine acceptance. This study aims to contribute to the ongoing reflections on tools and indicators of vaccine hesitancy by providing results of a knowledge, attitudes and beliefs (KAB) survey conducted among parents.

Methods: Data were collected in 2014 through a computer-assisted telephone interview survey administered to a sample of parents of children aged between 2 months and 17 years of age.

Results: The majority of the 589 parents included in the analyses agreed on the importance of vaccination to protect their children’s health and to prevent the spread of diseases in the community. The majority of the parents (81%) reported that their child had received all doses of recommended vaccines and 40% of parents indicated having hesitated to have their child vaccinated. Fear of adverse events and low perceived vulnerability of the child or severity of the disease were the most frequent reasons mentioned by these vaccine-hesitant parents. In multivariate analyses, KAB items remaining significantly associated both with an incomplete vaccination status of the child and parents’ vaccine hesitancy were: not thinking that it is important to have the child vaccinated to prevent the spreading of diseases in the community; not trusting the received vaccination information and having felt pressure to have the child vaccinated.

Discussion: Further researches will be needed to better understand when, how and why these beliefs are formed in order to prevent the onset of vaccine hesitancy.

## Introduction

Quebec (Canada) routine vaccination schedule for children and teens includes vaccines protecting against diphtheria, tetanus, whooping cough, measles, mumps, rubella, chicken pox, poliomyelitis, hepatitis B, flu (in autumn), and infections from Hib, pneumococcus, rotavirus, meningococcus C, and human papillomavirus (for girls). Vaccines are provided at no cost and more than 75% of vaccinated children aged 0-4 years are vaccinated by public health nurses in community health services clinics.^1^ Older children are vaccinated through school-based programs that are also under the responsibility of public community health services clinics. Vaccination is not mandatory in Quebec. The results of the 2014 population-based childhood vaccine coverage survey showed that 80% of one-year-old children and 71% of two-years-old children had received all doses of recommended vaccines.^1^ In 2013, 78% of girls in Grade 4 received the 2 recommended doses of the HPV vaccine^2^ and 86% of boys and girls in Grade 4 received the 2 recommended doses of the hepatitis B vaccine.^3^ Although these vaccine uptake rates are high, the objective to vaccinate 95% of children is still not reached. Studies in Quebec have highlighted that, despite publicly funded vaccination programs, access to vaccination services was sometimes problematic and important delays were observed in obtaining appointments for childhood vaccination.^4^^,^ 5 ^,^ 6 Results of other studies have shown that parental vaccine hesitancy also has a negative impact on vaccine uptake rates.^1^^,^ 7 ^,^ 8

The WHO Strategic Advisory Group of Experts (SAGE) Working Group on Vaccine Hesitancy defined vaccine hesitancy as “delay in acceptance or refusal of vaccines despite availability of vaccine services.”^9^ According to this group, the scope of vaccine hesitancy includes instances where “vaccine acceptance in a specific setting is lower than would be expected, given the availability of vaccination services.”^9^ A vaccine-hesitant person can delay, be reluctant (but still accept), or refuse one, some or all vaccines.^10^

Although increasingly used, the term vaccine hesitancy has been criticized as being an “ambiguous notion with an uncertain theoretical background”.^11^ The fact that vaccine hesitancy encompasses a heterogeneous group of individuals with diverse attitudes and behaviors makes the operationalization of this concept challenging.^10^ Although survey instruments have been developed and validated in the US^12^^,^ 13, these tools might not be adapted in other contexts.^14^ This study aims to contribute to the ongoing reflections on tools and indicators of vaccine hesitancy by providing results of a survey on knowledge, attitudes and beliefs (KAB) of parents in Quebec (Canada). We have compared KAB of parents according to: 1) self-reported vaccination status of their child and 2) hesitation during the vaccination decision.

## Methods

Data were collected through the 2014 Quebec study on vaccination against seasonal influenza and pneumococcal infections, a biennial repeated cross-sectional study in the province of Quebec, Canada. This paper presents the results pertaining to parents’ KAB about vaccination; the full report can be found elsewhere.^15^

Data were collected through a computer-assisted telephone interview survey conducted between March 18 and April 28 2014. Stratified random digit dialing (RDD) was used to select a geographically representative sample of the general population, including parents or caregivers of children aged between 2 months and 17 years of age. Households and respondents were both selected randomly. Parents with more than one child aged between 2 months and 17 years of age were asked to answer the questions for their youngest child. The interviewers asked the first name (or nickname) of that child, which was inserted in the question (e.g. it is important that you have *Juliet* vaccinated to protect her against diseases). Prior to each interview, verbal informed consent was obtained from the participant and interviews lasted in average 12.5 minutes. The study protocol was approved by the Research Ethics Board of the Research Center of the CHU de Québec - Université Laval. Polling firm has rigorous policies for ensuring data confidentiality and the research team received a de-identified database. Data were aggregated for analysis, and results are presented in order to be unable to identify individual responses.

Parents were asked 13 questions to measure their KAB about vaccination using a 5-point scale ranging from “Strongly agree” to “Strongly disagree”, and including “I do not know.” Self-reported vaccination status of the child was assessed for all participants using the question: With the exception of the common flu and H1N1 vaccines, since birth, has <*NAME*> had…? Following answers were proposed by the interviewer: 1- all the vaccines recommended in the regular Quebec vaccination program; 2- some vaccines only; 3- no vaccines. Children were considered up-to-date (UTD) when parents answered 1 and they were considered non-UTD when parents answered 2 or 3. Respondents who did not answer this question or who answered “I don’t know,” were excluded from the analyses. To assess vaccine hesitancy, parents were asked “Have you ever hesitated to have <*NAME*> vaccinated?” with the possibility to answer “Yes”, “No”, “I do not know”. Parents who answered “Yes” to that question were considered as being vaccine-hesitant, while others were not. All vaccine-hesitant parents were questioned about the main reason why they have hesitated (open-ended question) and what decision they have finally made regarding their child’s vaccination (four choices were possible: 1-the child has received all vaccines when due; 2- some vaccines were delayed, but the child finally has received all vaccines; 3- some vaccines were refused, but the child received some others; and 4- the child did not receive any vaccines). Vaccine-hesitant parents who accepted to give vaccines to their child (choices 1-2-3) were then asked about the main reasons for having finally accepted some or all vaccines.

The development of the survey questionnaire was informed by a literature review and expert consultations. The questionnaire was pilot tested by telephone with 10 participants to ensure clarity and appropriateness of the survey questions. Minor adjustments were made.

Expansion weights were assigned in order to ensure that the results were representative of the target population by adjusting for disproportionate sampling and non-response bias. Weighting was applied to each respondent in the sample based on socio-demographic characteristics drawn from the answers of respondents who agreed to participate but who were not eligible because of quotas already attained as well as from census data. Comparison of the answers to the KAB questions between parents of UTD or non-UTD children and between vaccine-hesitant and non-vaccine-hesitant parents were performed using Rao-Scott’s Chi-square test. To investigate which items predicted non-UTD vaccine status and parents’ vaccine hesitancy, multivariate analyses by weighted logistic regression were run while adjusting for parents’ sociodemographic characteristics (number of people in the household, education level, age and sex of the respondent). All 13 KAB items were included in the analyses. For each KAB item, answers have been dichotomized (“Strongly agree and “Somewhat agree” vs. “Strongly disagree” and “Somewhat disagree”), with the “I don’t know” answers systematically regrouped with the reference category. Items were sequentially tested and retained when their p-value was <0.05. All statistical analyses were performed using SAS statistical software version 9.3.

## Results

The overall response rate for the 2014 Quebec study on vaccination against seasonal influenza and pneumococcal infections was 35%. A total of 601 parents were interviewed, of these, 12 were excluded because the child’s immunization status was missing, leaving 589 parents for analysis. Respondents’ characteristics are shown in Table 1.

**Table 1. Sociodemographic characteristics of the respondents:** ^a^ 3 missing answers; ^b^ 2 missing answers.

Characteristics	n	Weighted %
Respondents' sex		
Male	206	49.2
Female	383	50.8
Respondents' age		
18-34 yrs	176	39.4
35-44 yrs	250	42.8
45-49 yrs	92	12.6
50 yrs and over	71	5.2
Children's age^a^		
0-4 yrs	195	40.2
5-17 yrs	361	59.8
Number of people in the household		
1-4	454	72.0
5 and more	135	28.0
Respondent's highest level of education^b^		
High scholl or less	159	44.0
College	198	22.4
University	230	33.6
Self-reported status of the child		
Up-to-date (UTD)	478	80.4
Non-UTD	111	19.6

A total of 478 (80.4%, [95% CI: 72.5–88.4]) children were declared to be UTD (Table 1). Overall, 218 parents (40.2%; [95% CI: 30.7–49.6]) said that they have hesitated to have their child vaccinated and most of them hesitated for some vaccines only (n=192). Fifty-eight percent (58.2%, [95% CI: 42.9–73.5%]) of children of vaccine-hesitant parents were UTD compared to 95.3% [95% CI: 91.2–99.4%] of children of non-vaccine-hesitant parents (p < 0.0001). Vaccines most frequently reported by vaccine-hesitant parents were: the influenza vaccine (n=99), the varicella vaccine (n=35), the HVP vaccine (n=28), and the rotavirus vaccine (n=20) (data not shown in Table).

Parents’ beliefs about vaccination according the vaccination status of the child and presence of vaccine hesitancy are reported in Table 2. In univariate analysis, many statistically significant differences were found between KAB of parents of UTD and non-UTD children. Parents of UTD children were significantly more likely than parents of non-UTD children to trust the information they receive on vaccination (p=0.023). In addition, more parents of UTD children considered that it is important that they have their child vaccinated both to prevent him/her against diseases (p=0.0111) and to prevent the spread of diseases in the community (p=0.006) when compared to parents of a non-UTD child. Parents of non-UTD children were significantly more likely to consider that children are receiving too many vaccines (p=0.0242), to believe that vaccines could weaken the immune system (p=0.0334) and to report feeling pressure to have their child vaccinated (p=0.0105). There were no differences between parents of UTD and non-UTD children for other questions: over 80% of parents considered that vaccines can prevent diseases and almost all parents were comfortable asking questions to doctors or nurses in regard to vaccination and 77% considered that diseases prevented by vaccines are serious. About one third indicated being fearful about vaccines. The same differences found between KAB of parents of UTD and non-UTD children were also found for vaccine-hesitant and non-vaccine-hesitant parents. Moreover, vaccine-hesitant parents were significantly more likely than non-vaccine-hesitant parents to report being fearful about vaccines (p=0.0111).

**Table 2. Participants' beliefs regarding vaccination, according parental self-report of the vaccination status of the child and vaccine hesitancy (%):** ^£^ Strongly agree and Somewhat agree; ^†^ Parental self-report of the vaccination status of the child, UTD = all vaccines, Non-UTD = only some vaccines/no vaccines; ^¥ ^Parental vaccine hesitancy level (Have you ever hesitated to have <*NAME*> vaccinated?), VH Parents = yes, Non-VH Parents = no; ^*^ p<0.05; ^**^ p<0.01.

Survey items		Vaccination status of the child		Parents' vaccine hesitancy	
	Total in Agreement^£ ^% [95% CI]	Total in Agreement^£ ^UTD % [95% CI]	Total in Agreement^£ ^Non-UTD^†^ % [95% CI]	Total in Agreement^£ ^VH Parents % [95% CI]	Total in Agreement^£ ^Non-VH Parents^¥^ % [95% CI]
You trust the vaccination information that you receive.	83.2 [75.2–91.2]	90.0 [84.0–96.0]	55.2^*^ [31.6–78.8]	66.6 [50.8–82.4]	94.3^** ^[89.9–98.7]
Vaccines can prevent diseases.	87.4 [80.3–94.5]	88.4 [80.9–96.0]	83.2 [64.7–100.0]	85.7 [73.2–98.3]	88.5 [80.2–96.8]
The diseases prevented by vaccines are serious.	77.0 [67.8–86.2]	79.1 [69.7–88.6]	68.2 [42.9–93.6]	73.8 [57.7–89.5]	79.3 [68.3–90.3]
You are comfortable asking doctors or nurses about vaccination.	99.1[98.4–99.8]	99.2 [98.4–100.0]	98.6 [96.9–100.0]	98.9 [97.8–99.9]	99.3 [98.3–100.0]
A good lifestyle, such as eating a healthy diet, can eliminate the need for vaccination.	43.4 [33.8–53.0]	39.5 [29.3–49.7]	59.4 [38.2–80.5]	53.3 [38.5–68.1]	36.8 [25.4–48.2]
Relying on alternative medicine like chiropractic, homeopathy or naturopathy can eliminate the need for vaccination.	20.9 [13.7–28.2]	18.2 [10.6–25.6]	32.3 [12.0–52.6]	24.0 [11.3–36.7]	18.9 [10.5–27.3]
Today, children are given too many vaccines.	37.7 [28.3–47.1]	31.8 [22.3–41.2]	62.1^*^ [41.3–82.8]	51.5 [36.4–66.6]	28.4^* ^[17.7–39.1]
You believe that vaccines run the risk of weakening the immune system.	31.6 [22.7–40.5]	25.9 [17.4–34.3]	55.0^*^ [32.9–77.1]	43.7 [28.5–59.0]	23.4^* ^[14.2–32.7]
Generally speaking, you are fearful about vaccines.	33.3 [25.1–41.5]	31.9 [22.8–41.0]	39.2 [19.2–59.2]	46.6 [32.0–61.2]	24.4^*^ [14.7–34.2]
Generally speaking, the people around you are in favour of vaccination.	81.6 [73.7–89.4]	85.7 [78.7–92.7]	64.7 [40.9–88.6]	73.7 [58.8–88.5]	86.8 [79.0–94.6]
It is important that you have your child vaccinated to protect him/her against diseases.	90.3 [83.6–97.1]	98.2 [97.3–99.1]	58.0^*^ [34.0–82.0]	77.8 [62.9–92.7]	98.7^** ^[97.9–99.5]
It is important that you have your child vaccinated to prevent the spreading of diseases in your community.	86.2 [78.9–93.5]	94.5 [90.8–98.3]	51.9^**^ [28.9–75.0]	71.3 [56.0–86.7]	96.2^** ^[94.0–98.3]
You have already felt pressure from people close to you or from society to have your child vaccinated.	35.0 [25.7–44.4]	28.2 [18.7–37.7]	63.3^*^ [42.3–84.2]	65.5 [52.3–78.7]	14.6^** ^[6.4–22.9]

Among the 218 vaccine-hesitant parents, the main reasons for having hesitated to vaccinate their child were collected in an open-ended question (Table 3). Fear of adverse events and low perceived vulnerability of the child or severity of the disease were the most frequent reasons mentioned by these vaccine-hesitant parents.

**Table 3. Main reasons for having hesitated to vacinate their child among the 218 vaccine-hesitant parents (%):** 

Reasons	n	Weighted %
Fear of adverse events	69	36.0
Low perception of vulnerability/severity of the disease	60	30.3
Doubts about vaccines	39	14.3
Influence of information on vaccination	19	7.0
Mistrust in general	7	4.5
Preference for other modes of prevention	9	2.8
Lack of knowledge/information	6	1.1
Other	9	4.2

Less than half of the 218 vaccine-hesitant parents (n=99), finally have accepted all vaccines when due and around 10% (n=27) have accepted all vaccines, but on a delayed schedule. More than one third of the vaccine-hesitant parents (n=87) have accepted some vaccines but refused others and a minority (n=5) have refused all vaccines. Among the 213 vaccine-hesitant parents who finally have accepted to give at least some vaccines to their child, 208 gave a reason for their decision. The main reasons reported were the protection of the child (n=92), having received a recommendation to vaccinate or more information about vaccination (n=26), trusting the recommendations (n=26) and social pressure to do so (n=15).

Results of the multivariate analyses are shown in Table 4. The modeled probabilities are for the non-UTD vaccine status of the children and the parents’ vaccine hesitancy. After adjustments, three items were both associated with an incomplete vaccination status of the child and parent vaccine hesitancy : not thinking that it is important to have the child vaccinated to prevent the spreading of diseases in the community, not trusting the received vaccination information and having felt pressure to have the child vaccinated. Not thinking that it is important to have the child vaccinated to protect him/her against diseases and believing that children are given too many vaccines were associated with incomplete vaccination status of the child. The items “The diseases prevented by vaccines are serious” remained significantly negatively associated with both the non-UTD status of the child and presence of vaccine hesitancy. “Vaccines prevent diseases” was another item negatively associated with the child non-UTD status, while the item “You believe that vaccines run the risk of weakening the immune system”) remained significantly negatively associated with vaccine hesitancy.

In multivariate analyses, the female respondent’s sex was significantly associated with a self-reported incomplete vaccine status for the child (adjusted OR=4.4, [95% CI: 1.9–10.0]; p=0.0005) as well as the respondent’s age of 45 years or over (adjusted OR=4.8, [95% CI: 1.8–12.9]; p=0.0018) and living in a household including 5 persons or more (adjusted OR=6.1, [95% CI: 2,1–18.2]; p=0.0011). No association was found for the parents’ vaccine hesitancy.

**Table 4. Regression analyses on the factors associated with non-UTD vaccine status of the child and parents' vaccine hesitancy:** ^1^ Taking into account other items in the model, and adjustments for the number of people in the household (continuous variable), education level, age (into 4 categories) and sex of the respondent.; * p<0.05; ** p<0.01.

Survey items	Non-UTD vaccine status of the child		Parents' vaccine hesitancy	
	Unadjusted OR	Adjusted OR [95% CI]	Unadjusted OR^1^	Adjusted OR^1^ [95% CI]
You trust the vaccination information that you receive. (disagree)	7.3^**^	3.3^**^ [1.3–8.5]	7.7^**^	8.8^**^[1.9–40.9]
Vaccines prevent diseases. (disagree)	1.5	0.3^*^[0.1–0.9]	1.3	0.9 [0.3–3.2]
The diseases prevented by vaccines are serious.(disagree)	1.9	0.3^*^ [0.1–0.9]	1.2	0.3^*^[0.1–1.0]
You are comfortable asking doctors or nurses about vaccination. (disagree)	1.9	0.3 [0.1–1.6]	1.6	0.8 [0.1–4.6]
A good lifestyle, such as eating a healthy diet, can eliminate the need for vaccination. (agree)	2.2	1.3 [0.5–3.3]	1.9	1.2 [0.4–3.4]
Relying on alternative medicine like chiropractic, homeopathy or naturopathy can eliminate the need for vaccination. (agree)	2.2	1.0 [0.4-2.7]	1.4	0.5 [0.2–1.5]
Today, children are given too many vaccines. (agree)	3.5^*^	3.1^* ^[1.1-8.9]	2.7^*^	3.1 [0.9–10.3]
You believe that vaccines run the risk of weakening the immune system. (agree)	3.5^*^	0.7 [0.2–2.3]	2.6^*^	0.3^*^ [0.1–0.8]
Generally speaking, you are fearful about vaccines. (agree)	1.4	0.5 [0.2–1.2]	2.7^*^	1.5 [0.6–3.8]
Generally speaking, the people around you are in favour of vaccination. (disagree)	3.3	0.9 [0.2–3.8]	2.7	0.8 [0.2–3.1]
It is important that you have your child vaccinated to protect him/her against diseases. (disagree)	38.1^**^	10.0^** ^[2.7–37.7]	21.2^**^	3.6 [0.5–24.4]
It is important that you have your child vaccinated to prevent the spreading of diseases in your community. (disagree)	16.0^**^	7.6^**^ [2.5–22.8]	10.1^**^	4.5^* ^[1.1–19.1]
You have already felt pressure from people close to you or from society to have your child vaccinated. (agree)	4.4^**^	3.1^**^ [1.2–7.8]	11.1^**^	11.1^**^ [4.9–25.4]

## Discussion

Vaccination remains one of the most important public health achievements worldwide. Despite the overall success of vaccination programs, it is often argued that public confidence in vaccines is decreasing.^16^^,^ 17 ^,^ 18 Even people who accept to be vaccinated or to have their child vaccinated can still have serious doubts and worries and be considered as vaccine-hesitant.^19^^,^ 20 This latter group is an important target for vaccination promotion interventions as they are “at-risk” of stopping vaccinating, but are more receptive to public health’s and healthcare providers’ messages than outright refusers.^21^

This study presents findings on the prevalence of vaccine hesitancy in a large and representative sample of parents in Quebec (Canada). Similar studies done in the United States have shown that around one third of parents could be considered as vaccine-hesitant.^12^^,^ 18 Our study shows similar figures, with 40% of the parents saying that they have already hesitated to vaccinate their child. Although parents’ self-report vaccine hesitancy, measured in a single question, is an imperfect proxy, it was used in a previous study and showed that 33% of the 938 surveyed parents were vaccine-hesitant and that hesitation can translate into behaviors that affect vaccine coverage (with at least one vaccine postponed in greater proportions among vaccine-hesitant parents).^7^ Due to the retrospective design of the study, we cannot rule out the fact that some parents may not recall their attitudes at the time of the vaccination decision. However, the statistically significant association between parents’ report of hesitancy and their child vaccination status indicated that the two measures capture to some extent the complex nature of vaccine hesitancy.

Vaccine hesitancy among vaccine-hesitant parents was mostly associated with some vaccines: generated by the influenza, the varicella, the HPV and the rotavirus vaccines. Some of these vaccines might be perceived by parents as being against “mild” diseases, which could increase vaccine hesitancy compared to vaccines against diseases perceived as “serious” and life-threatening.^22^^,^ 23 Research has also shown that new vaccines (such as the HPV and the rotavirus vaccines) are more prone to vaccine hesitancy than “older” vaccines. For instance, in a recent pan-Canadian survey, half of the parents were concerned that new vaccines are not as safe as older vaccines and one-third felt that children today receive too many vaccines, even if nine out of ten of these parents indicated their child’s vaccination was UTD.^24^ The HPV vaccine has also been subject to some controversies in the media since the introduction of publicly funded program in Quebec in 2008, which could also partially explain the fact that this vaccine has generated more hesitancy.^25^ The decision to implement a publicly funded program for vaccination against HPV was strongly criticized in Quebec. Negative media coverage around the HPV vaccine safety and usefulness has been observed sporadically over the past few years. For instance, recently some social scientists were publicly asking for a moratorium on the program (http://www.ledevoir.com/societe/sante/451710/vaccination-contre-les-vph-appel-urgent-a-un-moratoire). It is also reassuring to note that the majority of the vaccine-hesitant parents finally have decided to accept to have their child vaccinated. Most of the vaccine-hesitant parents chose to vaccinate because of the desire to protect their child from vaccine-preventable diseases or because they had received a recommendation to vaccinate from their providers. The tremendous influence of healthcare providers’ recommendations on vaccine acceptance is well-documented in the literature.^26^^,^ 27 ^,^ 28 ^,^ 29 ^,^ 30 ^,^ 31 However, some of the vaccine-hesitant parents said they agreed to vaccinate because of feeling pressure to do so. This could be interpreted as a positive indication that the pro-vaccine social norm is effectively enhancing vaccine compliance in Quebec, but also as an indication that these parents are still vaccine-hesitant, despite having accepted the vaccines. The latter is the most likely option, as having felt pressure to vaccinate was also a statistically significant factor of incomplete vaccine status in multivariate analysis. Many studies have shown the important influence of social norms on parents’ vaccination decisions^32^^,^ 33 ^,^ 34 ^,^ 35 ^,^ 36 and many studies have also indicated that even parents who accept to have their child vaccinated can still have important doubts and concerns.^13^^,^ 24 ^,^ 37

Our study has also identified factors linked with vaccine hesitancy and vaccine delays or refusals. In addition to feeling pressure to vaccinate, the strongest factors associated with both incomplete vaccine status and parents’ vaccine hesitancy were parents’ perception of the usefulness of vaccines to protect their community against the spread of vaccine-preventable diseases and parents’ belief that diseases prevented by vaccines are not serious. Similarly, believing that children are receiving too many vaccines was also a factor associated with incomplete vaccine status whereas believing that vaccines run the risk of weakening the immune system was associated with parental vaccine hesitancy. Another factor associated with an incomplete vaccine status was to not believing that vaccines prevent diseases. All of these factors are different measures of parents’ perception of the utility of vaccination, which has been also identified as a determinant of vaccination decision in numerous studies.^33^^,^ 34 ^,^ 38 ^,^ 39 ^,^ 40 ^,^ 41 ^,^ 42

Finally, another significant factor was a lack of trust in the information received about vaccination. While public health role is to ensure that the public is well-informed on vaccination, this finding highlights an important barrier when addressing vaccine hesitancy: the fact that some vaccine-hesitant parents might not trust the information about vaccination that they received from public health or their healthcare providers. An extensive review of the literature, has also highlighted that vaccine hesitancy in the public was not due to people being uninformed or misinformed, but rather because of multiple forms of distrust (of doctors, of government sources, of pharmaceutical companies).^43^ The authors of this review have concluded that, among a post-deferential public, the credibility of the institutions that deliver the information about vaccination seems to matter more than the information in itself, showing the complexity of factors impacting trust in vaccination. The theme of trust and mistrust of the pharmaceutical industry, governments, health authorities, doctors and research have also been highlighted in studies targeting healthcare workers.^44^ In our survey, we did not measure the level of trust in different sources of information about vaccination. Finally, some sociodemographic characteristics of the parents were associated with the incomplete vaccine status of the child (female sex, older age and number of people in the household (5 or more) but not with vaccine hesitancy.

Some limitations of our study must be addressed. The vaccine uptake status of the child was based on parental self-reported data and might therefore be subject to recall bias, which could result in over- or under-estimations of coverage. As Quebec has no central immunization registry, information on vaccination coverage can only be obtained through surveys. While another Quebec survey, based on a written questionnaire conducted every two years, uses the child vaccination booklet to determine the child vaccine status, we did not systematically ask the parent to refer to the child vaccination booklet in our telephone survey. The overall response rate for the study was low 35% and reason for non-participation was not asked. Finally, as mentioned, we cannot exclude the potential of socially desirable responses. However, the fact that the interviews were conducted by a professional polling firm should have minimized this bias.

To conclude, vaccine hesitancy is complex and multidimensional, varying across time, places and vaccines.^9^ As shown by this study, vaccine coverage could not be used as the sole indicator to measure vaccine hesitancy as it does not account for parents who have significant doubts and concerns about vaccines but still follow the recommended schedule. The number of factors influencing vaccine acceptance is dynamic and evolves over time. The use of diverse types of data and measurements approaches is thus needed to be able to capture this ever-moving target.^45^ There is an important need to build good measures that can identify and monitor patterns of vaccine hesitancy over time in individuals and populations. In that sense, identifying the vaccine-hesitant who still vaccinate and differentiating acceptance from access issues is essential.^46^ The findings of this study indicate that vaccine hesitancy is prevalent among parents in Quebec and provide some information that negative beliefs around usefulness of vaccination and lack of trust in the information received on this topic are factors that are associated with partial, delayed or non-vaccination. Further researches will be needed to better understand when, how and why these beliefs are formed in order to prevent the onset of vaccine hesitancy.

## Competing interests

The authors have declared that no competing interests exist.

## References

[1] Boulianne N, Audet D, Ouakki M, Dubé E, De Serres G, Guay M. Enquête sur la couverture vaccinale des enfants de 1 an et 2 ans au Québec en 2014. Québec: Institut national de santé publique du Québec, 2015.

[2] Gravel G, Markowski F, Toth E, Venne S. Résultats de la campagne scolaire 2012-2013. Flash Vigie. 2013;8(7):1-2.

[3] Judd L, Markowski F, Toth E. Vaccination contre l'hépatite B en milieu scolaire - Campagne 2013. Flash Vigie. 2014;9(9).

[4] Boulianne N, Kiely M, Sauvageau C, Guay M, Gilca V. Avis du Groupe scientifique en immunisation sur les indicateurs des retards vaccinaux au Québec. Québec: Institut national de santé publique du Québec, 2011.

[5] Guay M, Gallagher F, Petit G, Ménard S, Clément P, Boyer G. Pourquoi les couvertures vaccinales chez les nourrissons de l'Estrie sont-elles sous-optimales? Sherbrooke: Centre de santé et de services sociaux - Institut universitaire de gériatrie de Sherbrooke, 2009.

[6] Landry M. Évaluation préliminaire des retards dans le calendrier de vaccination au Québec, rapport préparé pour le Groupe provincial sur les retards en immunisation. 2005.

[7] Guay M, Ghorbel M, Lemaire J, Cadieux E, Désilets J, Clément P, et al. Vaccine hesitation among Quebec parents of children aged from 2 months to 5 years. Poster presentation. 11th Canadian Immunization Conference, Ottawa, December 2-4, 2014.

[8] Dubé E, Vivion M, Sauvageau C, Gagneur A, Gagnon R, Guay M. "Nature does things well, why should we interfere?": vaccine hesitancy among mothers. Qual Health Res. 2015. Epub 2015/02/26. 10.1177/1049732315573207 25711847

[9] MacDonald NE. Vaccine hesitancy: definition, scope and determinants. Vaccine. 2015;33(34):4161-4. 10.1016/j.vaccine.2015.04.036 25896383

[10] Dubé E, Laberge C, Guay M, Bramadat P, Roy R, Bettinger J. Vaccine hesitancy: an overview. Hum Vaccin Immunother. 2013;9(8):1763-73. 10.4161/hv.24657 PMC3906279 23584253

[11] Peretti-Watel P, Larson HJ, Ward JK, Schulz WS, Verger P. Vaccine hesitancy: clarifying a theoretical framework for an ambiguous notion. PLoS Curr. 2015;7. 10.1371/currents.outbreaks.6844c80ff9f5b273f34c91f71b7fc289 PMC4353679 25789201

[12] Opel DJ, Taylor JA, Zhou C, Catz S, Myaing M, Mangione-Smith R. The relationship between parent attitudes about childhood vaccines survey scores and future child immunization status: a validation study. JAMA Pediatr. 2013;167(11):1065-71. 10.1001/jamapediatrics.2013.2483 PMC4957941 24061681

[13] Gust DA, Darling N, Kennedy A, Schwartz B. Parents with doubts about vaccines: which vaccines and reasons why. Pediatrics. 2008;122(4):718-25. 10.1542/peds.2007-0538 18829793

[14] Larson HJ, Jarrett C, Schulz WS, Chaudhuri M, Zhou Y, Dube E, et al. Measuring vaccine hesitancy: the development of a survey tool. Vaccine. 2015;33(34):4165-75. 10.1016/j.vaccine.2015.04.037 25896384

[15] Dubé E, Gagnon D, Zhou Z, Guay M, Boulianne N, Sauvageau C, et al. Enquête québécoise sur la vaccination contre la grippe saisonnière et le pneumocoque, 2014. Québec: Institut national de santé publique du Québec, 2015.

[16] Black S, Rappuoli R. A crisis of public confidence in vaccines. Sci Transl Med. 2010;2(61):61mr1. 10.1126/scitranslmed.3001738 21148125

[17] Larson HJ, Cooper LZ, Eskola J, Katz SL, Ratzan S. Addressing the vaccine confidence gap. Lancet. 2011;378(9790):526-35. 10.1016/S0140-6736(11)60678-8 21664679

[18] Siddiqui M, Salmon DA, Omer SB. Epidemiology of vaccine hesitancy in the United States. Hum Vaccin Immunother. 2013;9(12):2643-8. 10.4161/hv.27243 PMC4162046 24247148

[19] Hilton S, Petticrew M, Hunt K."Combined vaccines are like a sudden onslaught to the body's immune system": parental concerns about vaccine "overload" and "immune-vulnerability". Vaccine. 2006;24(20):4321-7. 10.1016/j.vaccine.2006.03.003 16581162

[20] Kennedy A, Lavail K, Nowak G, Basket M, Landry S. Confidence about vaccines in the United States: understanding parents' perceptions. Health Affair. 2011;30(6):1151-9. 10.1377/hlthaff.2011.0396 21653969

[21] Leask J. Target the fence-sitters. Nature. 2011;473(7348):443-5. 10.1038/473443a 21614055

[22] MacDonald NE, Smith J, Appleton M. Risk perception, risk management and safety assessment: what can governments do to increase public confidence in their vaccine system? Biologicals. 2012;40(5):384-8. 10.1016/j.biologicals.2011.08.001 21993306

[23] Brewer NT, Chapman GB, Gibbons FX, Gerrard M, McCaul KD, Weinstein ND. Meta-analysis of the relationship between risk perception and health behavior: the example of vaccination. Health Psychol. 2007;26(2):136-45. 10.1037/0278-6133.26.2.136 17385964

[24] Ekos Research Associates Inc. Survey of parents on key issues related to immunization. Ottawa: Public Health Agency of Canada, 2011.

[25] Feinberg Y, Pereira JA, Quach S, Kwong JC, Crowcroft NS, Wilson SE, et al. Understanding public perceptions of the HPV vaccination based on online comments to Canadian news articles. PLoS One. 2015;10(6):e0129587. 10.1371/journal.pone.0129587 PMC4460033 26053866

[26] Clark SJ, Cowan AE, Wortley PM. Influenza vaccination attitudes and practices among US registered nurses. Am J Infect Control. 2009;37(7):551-6. 10.1016/j.ajic.2009.02.012 19556035

[27] Hollmeyer HG, Hayden F, Poland G, Buchholz U. Influenza vaccination of health care workers in hospitals: a review of studies on attitudes and predictors. Vaccine. 2009;27(30):3935-44. 10.1016/j.vaccine.2009.03.056 19467744

[28] Posfay-Barbe KM, Heininger U, Aebi C, Desgrandchamps D, Vaudaux B, Siegrist CA. How do physicians immunize their own children? Differences among pediatricians and nonpediatricians. Pediatrics. 2005;116(5):e623-33. 10.1542/peds.2005-0885 16263976

[29] Katz-Sidlow RJ, Sidlow, R. A look at the pediatrician as parent: experiences with the introduction of varicella vaccines. Clin Pediatr (Phila). 2003;42(7):635-40. 10.1177/000992280304200710 14552523

[30] Zimmerman RK, Bradford BJ, Janosky JE, Mieczkowski TA, DeSensi E, Grufferman S. Barriers to measles and pertussis immunization: the knowledge and attitudes of Pennsylvania primary care physicians. Am J Prev Med. 1997;13(2):89-97. 9088444

[31] Connors CM, Miller NC, Krause VL. Universal hepatitis B vaccination: hospital factors influencing first-dose uptake for neonates in Darwin. Aust N Z J Public Health. 1998;22(1):143-5. 10.1111/j.1467-842x.1998.tb01159.x 9599867

[32] Brunson EK. The impact of social networks on parents' vaccination decisions. Pediatrics. 2013;131(5):e1397-404. 10.1542/peds.2012-2452 23589813

[33] Brown KF, Kroll JS, Hudson MJ, Ramsay M, Green J, Long SJ, et al. Factors underlying parental decisions about combination childhood vaccinations including MMR: a systematic review. Vaccine. 2010;28(26):4235-48. 10.1016/j.vaccine.2010.04.052 20438879

[34] Tickner S, Leman PJ, Woodcock A. Factors underlying suboptimal childhood immunisation. Vaccine. 2006;24(49-50):7030-6. 10.1016/j.vaccine.2006.06.060 16890330

[35] Hilton S, Petticrew M, Hunt K. Parents' champions vs. vested interests: who do parents believe about MMR? A qualitative study. BMC Public Health. 2007;7:42. 10.1186/1471-2458-7-42 PMC1851707 17391507

[36] Petts J, Niemeyer S. Health risk communication and amplification: learning from the MMR vaccination controversy. Health, Risk & Society. 2004;6(1):7-23.

[37] Kennedy A, Basket M, Sheedy K. Vaccine attitudes, concerns, and information sources reported by parents of young children: results from the 2009 HealthStyles survey. Pediatrics. 2011;127 (Suppl 1):S92-9. 10.1542/peds.2010-1722N 21502253

[38] Falagas ME, Zarkadoulia E. Factors associated with suboptimal compliance to vaccinations in children in developed countries: a systematic review. Curr Med Res Opin. 2008;24(6):1719-41. 10.1185/03007990802085692 18474148

[39] Mills E, Jadad AR, Ross C, Wilson K. Systematic review of qualitative studies exploring parental beliefs and attitudes toward childhood vaccination identifies common barriers to vaccination. J Clin Epidemiol. 2005;58(11):1081-8. 10.1016/j.jclinepi.2005.09.002 16223649

[40] Sturm LA, Mays RM, Zimet GD. Parental beliefs and decision making about child and adolescent immunization: from polio to sexually transmitted infections. J Dev Behav Pediatr. 2005;26(6):441-52. 10.1097/00004703-200512000-00009 16344662

[41] Quadri-Sheriff M, Hendrix KS, Downs SM, Sturm LA, Zimet GD, Finnell SM. The role of herd immunity in parents' decision to vaccinate children: a systematic review. Pediatrics. 2012;130(3):522-30. 10.1542/peds.2012-0140 22926181

[42] Roberts KA, Dixon-Woods M, Fitzpatrick R, Abrams KR, Jones DR. Factors affecting uptake of childhood immunisation: a Bayesian synthesis of qualitative and quantitative evidence. Lancet. 2002;360(9345):1596-9. 10.1016/S0140-6736(02)11560-1 12443615

[43] Yaqub O, Castle-Clarke S, Sevdalis N, Chataway J. Attitudes to vaccination: a critical review. Soc Sci Med. 2014;112:1-11. 10.1016/j.socscimed.2014.04.018 24788111

[44] European Centre for Disease Prevention and Control. Vaccine hesitancy among healthcare workers and their patients in Europe - A qualitative study. Stockholm: ECDC, 2015.

[45] Larson H, Schulz W. The state of vaccine confidence [Internet]. 2015. Available from: http://www.vaccineconfidence.org/The-State-of-Vaccine-Confidence-2015.pdf.

[46] Leask J, Willaby HW, Kaufman J. The big picture in addressing vaccine hesitancy. Hum Vaccin Immunother. 2014;10(9):1-3. 10.4161/hv.29725 PMC4975059 25483479

